# The Influence of Obesity on Different Genders in Patients with Obstructive Sleep Apnea

**DOI:** 10.1155/2014/487215

**Published:** 2014-07-14

**Authors:** Kuo-Tung Huang, Chien-Hung Chin, Chia-Cheng Tseng, Huang-Chih Chang, Yung-Che Chen, Chin-Chou Wang, Meng-Chih Lin, Hsin-Ching Lin, Mao-Chang Su

**Affiliations:** ^1^Division of Pulmonary and Critical Care Medicine, Department of Internal Medicine, Kaohsiung Chang Gung Memorial Hospital and Chang Gung University College of Medicine, Kaohsiung 83301, Taiwan; ^2^Sleep Center, Kaohsiung Chang Gung Memorial Hospital and Chang Gung University College of Medicine, Kaohsiung 83301, Taiwan; ^3^Department of Respiratory Care, Chang Gung University of Science and Technology, Chiayi Campus, Chiayi 61363, Taiwan; ^4^Department of Otolaryngology, Kaohsiung Chang Gung Memorial Hospital and Chang Gung University College of Medicine, Kaohsiung 83301, Taiwan

## Abstract

Obesity is considered to be a major contributing factor to obstructive sleep apnea (OSA); however, there is limited evidence with regard to gender predominance. We analyzed 2345 patients (339 females) in correlation with body mass index (BMI) and OSA severity. Male AHIs were significantly higher than female AHIs in each BMI group. As the BMI increased, the AHI increased in both males and females, and this trend was more obvious in males. For BMI-matched male and female patients with OSA, the severity of OSA was higher in males. As BMI increased, the severity of OSA increased more obviously in males. Our findings suggest that increased body fat contributes to the pathogenesis of OSA more in males than in females and that obesity plays a more significant role in contributing to OSA in male patients.

## 1. Introduction

Several factors contribute to the development of obstructive sleep apnea (OSA), of which obesity is the most common. One way by which obesity predisposes to OSA is through pharyngeal fat deposition, which increases peripharyngeal soft tissue pressure resulting in a narrower and more collapsible upper airway (UA) [1]. A higher body mass index (BMI) is closely related to the incidence and severity of OSA [2], and body weight loss induced either by diet control or by barometric surgery has been shown to mitigate OSA [3]. Accordingly, body weight gain worsens OSA and the degree of obesity is proportionally associated with the severity of OSA. Body weight loss should therefore be advised in clinical practice, even though only a few patients will be able to do so.

The prevalence of OSA in men is at least twice that in women [4–6]; however the mechanisms responsible for this are not well understood. Factors that influence UA collapsibility and therefore susceptibility to OSA may differ between men and women. For example, the longer collapsible pharyngeal segment [7] and greater increase in UA resistance after sleep onset in men than in women [8] may contribute to a greater UA collapsibility in men. It is highly possible that the male UA becomes more collapsible when peripharyngeal soft tissue pressure is increased, particularly when there is excessive peripharyngeal fat deposition such as in obese subjects. However, only a limited number of studies have reported on the extent to which this predisposing factor contributes to OSA, particularly with regard to gender. In the present study, we analyzed the variables obtained from our sleep studies to investigate whether an increased BMI is correlated with a greater susceptibility to OSA more in men than in women.

## 2. Methods

### 2.1. Patients

From March 2002 to March 2009, all adult subjects (aged 18 years or more) receiving baseline polysomnographic studies in our sleep laboratory were included. Patients with major cardiopulmonary disorders needing aggressive treatment or a history of previous major UA surgery were excluded. If a patient had undergone more than one baseline sleep study, only the variables obtained from the first study were used for analysis. All patients diagnosed with OSA (defined as an apnea-hypopnea index (AHI) ≥ 5/h determined by polysomnography accompanied by at least one symptom such as habitual snoring and excessive daytime sleepiness) were enrolled in the study.

### 2.2. Polysomnography

Body height, body weight, and BMI were measured prior to the overnight polysomnographic study. Subjective sleepiness was assessed using the Epworth Sleepiness Scale (ESS), an 8-point questionnaire that assesses a subject's tendency to fall asleep during various situations, where a higher score indicates increased sleepiness [9, 10]. The complete polysomnography examination included electroencephalography, electrooculography, chin and anterior tibial electromyography, respiratory effort detectors, nasal/oral flow sensors, and pulse oximetry and was performed using a standardized commercial device (Alice 4, Respironics, Georgia, USA).

All subjects completed their polysomnographic study with at least 4 hours of total sleep time as indicated by electroencephalography. Sleep stage scoring was done at 30-second intervals by experienced technicians according to the standard criteria [11]. Obstructive apnea was defined as a cessation of airflow for at least 10 seconds with the subject making an effort to breathe during apnea. Obstructive hypopnea was defined as an abnormal respiratory event with at least a 30% reduction in thoracoabdominal movement or airflow as compared to baseline, lasting for at least 10 seconds, with a greater than 4% oxygen desaturation. The AHI was defined as the total number of apneas and hypopneas per hour of electroencephalographic sleep.

### 2.3. Study Protocol

Both male and female patients with OSA were subdivided into 3 groups according to their BMI based on classical classification [12] as follows: nonoverweight patients (BMI < 24 kg/m^2^), overweight patients (24 kg/m^2^ ≤ BMI < 27 kg/m^2^), and obese patients (BMI ≥ 27 kg/m^2^). AHI and other parameters obtained from the sleep studies were compared between both genders in each group.

### 2.4. Statistical Analysis

All data are expressed as mean ± SD unless otherwise stated. One-way ANOVA was used to compare values between men and women in the three groups, followed by post hoc Bonferroni and Scheffe tests as appropriate, and the linear trend of AHI, arousal index, mean oxygen saturation, lowest oxygen saturation, and other variables were assessed by a polynomial test. If the variables were nonnormally distributed, the Mann-Whitney *U* test was used for comparison. In addition, the linear trend of the three groups between men and women was assessed by using general linear model univariate analysis. Propensity score matching analysis was also performed for statistical matching. Data analysis was done with commercial statistical analysis software packages (SPSS 13.0 and NCSS 2007), and a two-sided *P* value less than 0.05 was considered to be statistically significant.

## 3. Results

**Figure 1:**
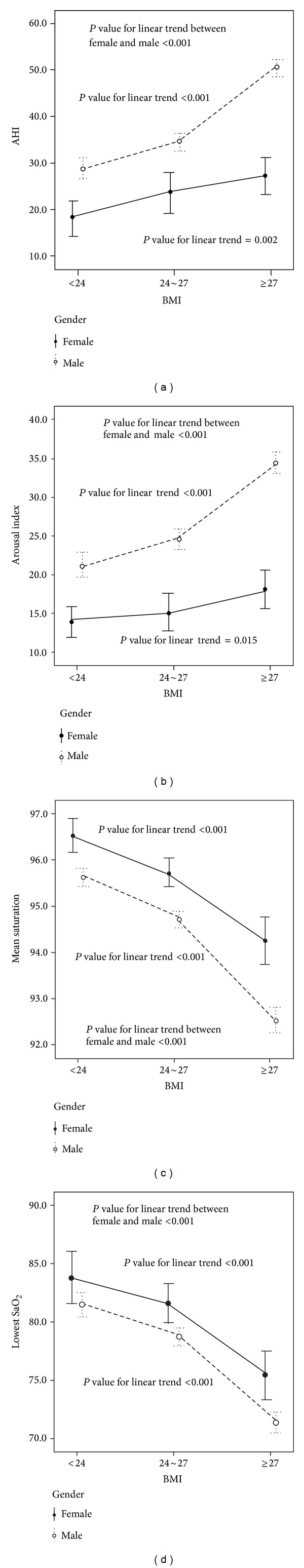
The trends of AHI, arousal index, mean saturation, and lowest saturation in both male and female patients with obstructive sleep apnea (OSA). As the BMI increased, the AHI and arousal index increased significantly, while the mean saturation and lowest saturation decreased significantly in both genders. These trends were significantly predominant in the male patients.

In total, 2006 male and 339 female ethnic Chinese patients with OSA diagnosed after the baseline polysomnographic studies were enrolled. The characteristics and polysomnographic variables are shown in Table 1. The female patients were older, but there were no significant differences in BMI and sleep efficiency between both genders. The ESS, AHI, arousal index, and desaturation index were significantly higher, while the mean saturation and lowest saturation were lower in the male patients than in the female patients. The male AHIs were significantly higher than those of the female patients in all three BMI groups (nonoverweight patients, 28.8 ± 21.1/h for males and 18.0 ± 17.7/h for females, *P* < 0.001; overweight patients, 34.3 ± 23.2/h for males and 23.2 ± 20.3/h for females, *P* < 0.001; obese patients, 50.3 ± 29.7/h for males and 27.1 ± 25.2/h for females, *P* < 0.001). In addition, as the BMI increased, the AHI and arousal index increased significantly, while the mean saturation and lowest saturation decreased significantly in both genders. These trends were more predominant in the male patients than in the female patients (Figure 1).

**Figure 2:**
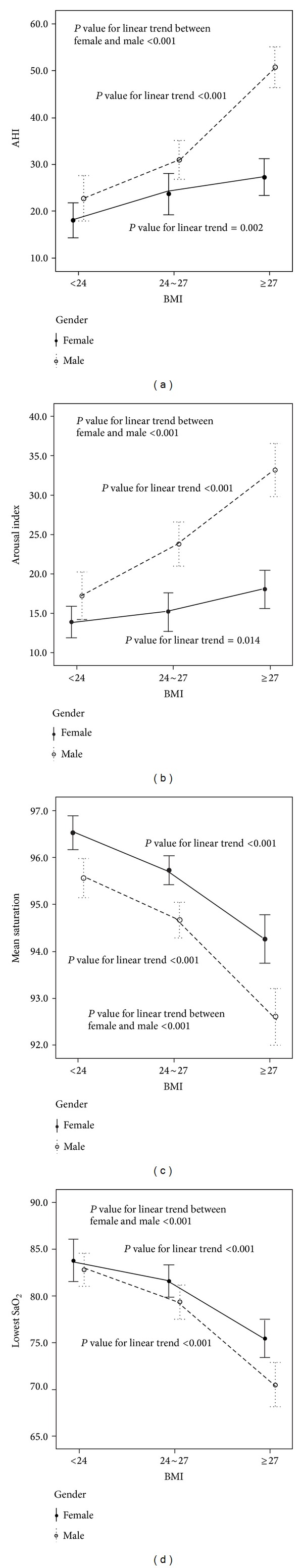
The trends of AHI, arousal index, mean saturation, and lowest saturation in both BMI- and age-matched male and female patients with OSA. As the BMI increased, the AHI and arousal index increased significantly, while the mean saturation and lowest saturation decreased significantly in both genders. These trends were significantly predominant in the male patients.

Propensity score matching analysis was used due to the female subjects being older. In total, 672 BMI- and age-matched patients (336 male and 336 female patients) were included in this analysis. As shown in Table 2, the ESS, AHI, arousal index, and desaturation index were higher, while the mean saturation and lowest saturation were lower in the male BMI- and age-matched patients compared to the female BMI- and age-matched patients. The male AHIs were again higher than the female AHIs in all BMI groups. Similarly, as the BMI increased, the AHI and arousal index increased significantly, while the mean saturation and lowest saturation decreased significantly in both genders. These trends were still more predominant in the age-matched male patients (Figure 2).

## 4. Discussion

In the present study, we found that the severity of OSA was higher in the male BMI-matched patients compared to the female BMI-matched patients. As the BMI increased, the severity of OSA increased more in the male than in the female patients. These findings suggest that an increased body weight contributes to OSA more in men than in women. In addition, obesity may play a more significant role in contributing to OSA in male patients.

Patients usually seek medical help and receive sleep studies due to sleep-disordered breathing (SDB), which includes primary snoring, upper airway resistance syndrome, and OSA. OSA is characterized by repeated total or partial pharyngeal collapse during sleep which leads to intermittent hypoxemia and sleep fragmentation. OSA not only results in excessive daytime sleepiness and impaired cognitive function, but also correlates significantly with cardiovascular morbidities such as stroke, coronary artery disease, acute myocardial infarction, congestive heart failure, and cardiac arrhythmias [13–15].

The prevalence rates of SDB (defined as an AHI ≥ 5/h) and OSA (defined as an AHI ≥ 5/h accompanied by at least one symptom such as habitual snoring and excessive daytime sleepiness) in the general population have been reported to be 20% [16] and 2~9% [17], respectively. The incidence of OSA has also been reported to be higher in males than in females, with a ratio of approximately 2 : 1 [17]. Similar findings have been reported in Asian patients. For example, the prevalence of SDB has been reported to be 27% in men and 16% in women, and the prevalence of OSA has been reported to be 4.5% in men and 3.2% in women in Korea [18].

The mechanisms responsible for the higher prevalence of OSA in men than in women are not well understood; however, neuromuscular and anatomical-mechanical factors that influence UA patency appear to be involved. With respect to neuromuscular factors, the results of various studies are inconsistent. Trinder et al. [8] demonstrated that, after sleep onset, there was a greater decrease in genioglossus muscle activity and a greater increase in UA resistance in men than in women, thereby increasing the susceptibility to UA occlusion. Popovic and White [19] also showed that awake genioglossal activity was significantly higher in healthy women than in healthy men. However, some studies have not demonstrated any differences in genioglossus muscle activity in healthy men and women during either wakefulness or sleep [7, 20, 21] or in UA muscle activation in response to inspiratory resistive loading [21]. Therefore, the contribution of differences in neural control of pharyngeal dilator muscles to the differences in susceptibility to OSA between men and women remains uncertain.

Regarding anatomical-mechanical factors, anatomical narrowing of the UA has been shown to play an important role in predisposing to OSA [22–24], and UA properties have been reported to be related to the presence and severity of OSA. For example, patients with OSA have been shown to have a narrower, more compliant, and more collapsible UA than subjects without OSA during both wakefulness and sleep [25–27].

Nevertheless, since women have smaller UA luminal dimensions than men [28], this cannot explain the lower prevalence of OSA in women than in men. In addition, no differences in UA resistance during wakefulness and sleep between healthy men and women have been reported [19, 29]. UA resistance is related to UA cross-sectional area and UA length. Although women have a narrower UA [28], and UA resistance is similar in both genders [19, 29], it appears that the collapsible segment of the UA is longer in men than in women [7]. This difference in UA length, which develops after puberty [30], is not a function of men being taller than women, since the male pharyngeal collapsible segment is still longer when normalized to height. However, a greater length of collapsible UA segment would increase the propensity for collapse. Therefore, based on these anatomical factors, pharyngeal collapsibility would appear to be greater in men than in women for a given negative pressure during inspiration. However, Rowley et al. [29] found no difference in critical closing pressure between healthy men and women during sleep. Thus, the extent to which differences in pharyngeal length between men and women influence the susceptibility to UA collapse is unclear.

Although there is no difference in UA collapsibility between healthy men and women [29], it is possible that UA collapsibility changes when UA conditions are altered and that such changes differ between genders. For example, men have been shown to be more vulnerable to resistive loading- and position-induced UA collapse than women [21, 31]. Su et al. reported that the male UA is more collapsible due to peripharyngeal fluid accumulation caused by rostral fluid displacement from the lower body [32]. Likewise, when the degree of fat deposition around the UA increases, it becomes more collapsible in men than in women. Obesity is a major contributing factor to OSA due to peripharyngeal fat accumulation accompanied by an increase in neck circumference. The increased fatty tissue surrounding the UA predisposes to OSA by decreasing the UA lumen caliber and increasing UA collapsibility [1]. Factors such as obesity and fluid overload influence UA collapsibility and therefore susceptibility to OSA in a different manner between men and women. Our findings support that obesity increases UA collapsibility and that this increase is greater in men than in women.

The association of obesity and OSA severity has also been demonstrated in longitudinal fashion. For example, a positive association between the severity of SDB and an increase in BMI over approximately 5 years has been reported [33]. In addition, body weight loss induced either by diet control or by barometric surgery has been shown to mitigate OSA [3]. It would be necessary to conduct a prospective study in a reciprocal fashion to see if body weight reduction diminishes OSA and whether this effect differs between men and women.

In general, Asian subjects have a smaller bony structure so that soft tissue pressure would increase more easily when fat accumulates around the UA, and a higher BMI has been shown to be a major predictive and risk factor for SDB in Chinese [34, 35] and Korean subjects [18]. Ip and coworkers reported that women with SDB had lower AHIs compared to men with similar BMIs [34, 35]. With a larger subject number, our study also demonstrated the severity of OSA in BMI-matched ethnic Chinese men and women. Nevertheless, this study is the first to show a trend of increased OSA severity associated with increased BMI in both genders, and this trend was more predominant in male than in female patients. Whether this result can be extrapolated to other ethnic populations requires further investigations.

The characteristics between both genders in this study were similar, except that the female patients were older. Several studies investigating gender differences reported similar findings [36–38]. It is possible that if we had enrolled more elderly male OSA subjects to match the ages of both the groups, the effect of obesity on male OSA may have been more pronounced. In addition, after propensity score matching analysis, higher AHI and arousal index and lower mean saturation and lowest saturation were still observed in the male patients. This enforces the concept that an increased BMI contributes to the severity of OSA more in men than in BMI- and age-matched women.

There are some limitations to this study. First, AHI was used to measure the severity of OSA and was then used in the statistical analysis. In patients with severe OSA, a long apnea period may result in decreased apnea/hypopnea counts in a certain period, thus leading to a lower AHI. However, AHI is the most widely used parameter to present the severity of OSA, even though more severe sleep disruption may be seen in milder OSA [39]. Furthermore, other parameters such as lowest saturation and mean desaturation were also correlated to OSA severity and showed significant differences between genders. Second, the conditions of fat deposition as well as other anthropometric parameters of the patients were not* shown since the data was collected retrospectively and some measures were missing*. It is possible that UA changes differ between genders in response to body fat deposition around the UA. Such changes may contribute to more favorable UA mechanics in women and the larger but more collapsible UA in men [28, 40]. However, there were a large number of subjects in this study, making the further individual anatomical and mechanical analysis infeasible. To avoid the influence of anatomic structural changes, the patients with a history of previous major UA surgery were excluded from this study.

In conclusion, the severity of OSA was higher in the male patients compared to the female patients with similar BMIs. As the BMI increased, the severity of OSA increased more significantly in males. Obesity played a more important role in contributing to OSA in men than in women. Mechanisms may exist in women which protect their UA from collapse during sleep, particularly when they are obese. Further investigations regarding gender differences in SDB may be warranted.

## Figures and Tables

**Table 1:** Characteristics and polysomnographic variables of the male and female patients with obstructive sleep apnea.

	Female (*n* = 339)	Male (*n* = 2006)	*P* value	95% confidence interval	
BMI (kg/m^2^)	27.8 ± 5.9	27.8 ± 4.4	0.869	−0.598	0.708
Age (years)	48.5 ± 12.2	45.6 ± 12.8	<0.001	1.475	4.407
ESS	9.4 ± 5.4	10.4 ± 5.2	0.001	−1.613	−0.397
AHI (/h)	23.7 ± 22.5	41.8 ± 28.1	<0.001	−20.711	−15.316
Sleep efficiency (%)	76.6 ± 16.6	77.8 ± 15.8	0.187	−3.072	0.600
Arousal index (/h)	16.2 ± 13.4	29.12 ± 21.2	<0.001	−15.256	−10.572
Desaturation index (/h)	24.4 ± 24.1	38.6 ± 28.0	<0.001	−17.098	−11.395
Mean SaO_2_ (%)	95.2 ± 2.8	93.7 ± 3.7	<0.001	1.173	1.843
Lowest SaO_2_ (%)	79.2 ± 11.9	75.4 ± 13.4	<0.001	2.454	5.255

Values are mean ± SD. BMI, body mass index; ESS, Epworth Sleepiness Scale; AHI, apnea-hypopnea index; SaO_2_, oxygen saturation.

**Table 2:** Characteristics and polysomnographic variables of the male and female patients with obstructive sleep apnea after propensity score matching analysis.

	Female (*n* = 336)	Male (*n* = 336)	*P* value	95% confidence interval	
BMI (kg/m^2^)	27.8 ± 5.9	27.8 ± 4.6	0.993	−0.802	0.795
Age (years)	48.6 ± 12.2	48.6 ± 12.7	0.990	−1.871	1.895
ESS	9.4 ± 5.4	10.7 ± 5.2	0.002	−2.099	−0.496
AHI (/h)	23.7 ± 22.5	39.7 ± 27.5	<0.001	−19.568	−11.952
Sleep efficiency (%)	76.6 ± 16.7	77.2 ± 15.3	0.602	−3.061	1.777
Arousal index (/h)	16.3 ± 13.4	27.6 ± 19.4	<0.001	−13.850	−8.779
Desaturation index (/h)	24.6 ± 24.1	37.0 ± 27.4	<0.001	−16.348	−8.526
Mean SaO_2_ (%)	95.2 ± 2.8	93.8 ± 3.3	<0.001	0.999	1.924
Lowest SaO_2_ (%)	79.2 ± 11.9	75.4 ± 13.6	<0.001	1.825	5.700

Values are mean ± SD. BMI, body mass index; ESS, Epworth Sleepiness Scale; AHI, apnea-hypopnea index; SaO_2_, oxygen saturation.

## Abbreviations

AHI: Apnea-hypopnea index
BMI: Body mass index
ESS: Epworth Sleepiness Scale
OSA: Obstructive sleep apnea
SaO_2_: Oxygen saturation
SDB: Sleep-disordered breathing
UA: Upper airway.
